# Epigenetic Pattern on the Human Y Chromosome Is Evolutionarily Conserved

**DOI:** 10.1371/journal.pone.0146402

**Published:** 2016-01-13

**Authors:** Minjie Zhang, Chuan-Chao Wang, Caiyun Yang, Hao Meng, Ikechukwu O. Agbagwa, Ling-Xiang Wang, Yingzhi Wang, Shi Yan, Shancheng Ren, Yinghao Sun, Gang Pei, Xin Liu, Jiang Liu, Li Jin, Hui Li, Yingli Sun

**Affiliations:** 1 Key Laboratory of Genomic and Precision Medicine, China Gastrointestinal Cancer Research Center, Beijing Institute of Genomics, Chinese Academy of Sciences, Beijing 100101, China; 2 University of Chinese Academy of Sciences, Beijing, 100049, China; 3 State Key Laboratory of Genetic Engineering and MOE Key Laboratory of Contemporary Anthropology, School of Life Sciences, Fudan University, Shanghai, 200433, China; 4 Department of Plant Science & Biotechnology, University of Port Harcourt, Port Harcourt, Nigeria; 5 Department of Urology, Shanghai Changhai Hospital, Second Military Medical University, Shanghai, 200433, China; 6 State Key Laboratory of Cell Biology, Institute of Biochemistry and Cell Biology, Shanghai Institutes for Biological Sciences, Graduate School of the Chinese Academy of Sciences, Chinese Academy of Sciences, Shanghai, 200031, China; 7 Shanghai Key Laboratory of Signaling and Disease Research, School of Life Science and Technology, Tongji University, Shanghai, 200092, China; 8 Key Laboratory of Genome Sciences and Information, Beijing Institute of Genomics, Chinese Academy of Sciences, Beijing, 100101, China; Harbin Medical University, CHINA

## Abstract

DNA methylation plays an important role for mammalian development. However, it is unclear whether the DNA methylation pattern is evolutionarily conserved. The Y chromosome serves as a powerful tool for the study of human evolution because it is transferred between males. In this study, based on deep-rooted pedigrees and the latest Y chromosome phylogenetic tree, we performed epigenetic pattern analysis of the Y chromosome from 72 donors. By comparing their respective DNA methylation level, we found that the DNA methylation pattern on the Y chromosome was stable among family members and haplogroups. Interestingly, two haplogroup-specific methylation sites were found, which were both genotype-dependent. Moreover, the African and Asian samples also had similar DNA methylation pattern with a remote divergence time. Our findings indicated that the DNA methylation pattern on the Y chromosome was conservative during human male history.

## Introduction

DNA methylation, which refers to as the covalent addition of a methyl group to the fifth carbon of cytosine (resulting in the production of 5-methylcytosine at CpG sites), is called the “fifth base” of the DNA code [1]. As an important type of epigenetic modification, DNA methylation plays essential roles in many biological processes, including gene regulation, mammalian development, X chromosome inactivation, and genomic imprinting [2–7]. Moreover, abnormal methylation modifications represent an important link to disease susceptibility, such as in Rett syndrome, monogenic disease, and cancer [8–11]. Previous studies showed that double knockout of the DNA methyltransferases DNMT1 and DNMT3a/3b in mice could result in defects in embryogenesis [12, 13]. Recently, a lot of research focused on the study of DNA methylation during mammalian development, reprogramming, and inheritance [14–16]. Several studies showed that the genome-wide DNA methylation underwent methylation reprogramming during early embryonic development [17–20].

However, whether DNA methylation can be stably passed from generation to generation like the genetic code is an open question remaining to be elucidated. Unfortunately, there are many obstacles to address this question in humans. First, DNA methylation pattern varies from tissue to tissue, which makes it difficult to select a standard for comparison [21]. Second, although quite a few population-specific DNA methylation patterns have been found, it is difficult to make a connection between these variations and certain populations or haplogroups [22]. Furthermore, epigenetic variations after recombination between sister chromatids also make epigenetic analysis difficult and complicated.

Recently, researchers found that more than 2000 differential methylation regions (DMRs) existed between ancient and modern human population [23]. Moreover, Radford *et al*. found that the germline DNA methylation of adult mice could be altered by the in utero nutritional environment of embryos, while the next generation would not be affected [24]. However, it is difficult to study epigenetic changes in more than 4 generations in one family pedigree.

Interestingly, the Y chromosome, unlike other chromosomes, is transferred between males and is only inherited from father to son. A lot of research work revealed important and unappreciated role of the Y chromosome of human [25–28]. Most regions on the Y chromosome remains comparable stability and purity after inheritance through multiple generations. Therefore, studying the methylation pattern on the Y chromosome could help overcome the above obstacles.

Single-nucleotide polymorphisms (SNPs) on the Y chromosome have been successfully utilized to reconstruct the Y chromosome phylogeny and to identify the divergence time of different lineages [29–34]. Through the detection of phylogenetically relevant SNPs within non-recombinant regions of the Y chromosome (NRY), we reconstructed the Y chromosome phylogeny of 72 donors [35–38]. The divergence time of these samples ranges from approximately 50 years to several thousand years ago (kya) [39]. Further, we compared their DNA methylation pattern on the Y chromosome.

Based on the above powerful tools and analysis, we found that DNA methylation pattern on the Y chromosome was conserved within family trees and haplogroups despite of a few variations within individual sample. We also revealed that there were some genotype-related polymorphic DNA methylation variations on the Y chromosome. The conserved methylation pattern within each haplogroup and the genotype-dependent polymorphic variations shared by all individuals in specific haplogroups indicated that the methylation pattern on the Y chromosome was stable during human history.

## Materials and Methods

### Sample collection and DNA extraction

To study the methylation pattern of different haplogroups, we collected whole blood samples from 72 male donors. Our study was approved by the Ethics Committee of Beijing Institute of Genomics, Chinese Academy of Sciences, School of Life Sciences, Fudan University, and the University of Port Harcourt. All individuals were adequately informed and signed an informed consent form before participating in the study. These samples were all collected based on deep-rooted pedigrees and the Y chromosome phylogenetic tree described in our previously published work. DNA was extracted from the whole blood using QIAamp DNA Mini Kit (QIAGEN, Hilden, Germany).

### Haplogroup analysis

In addition to the previously listed primers of the latest Y chromosome phylogenetic tree, we also designed several primers for newly typed SNPs [40, 41]. Overall, we tested more than 100 Y chromosome SNPs to establish the Y chromosome haplotype of 72 donors.

### Data generation

DNA samples were bisulfate-converted using a ZymoEZ DNA Methylation Kit (Zymo Research). After being amplified and enzymatically fragmented, the resulted fragments were purified by isopropanol precipitation and hybridized to the Infinium Human Methylation 450 BeadChip array (Illumina Inc., San Diego, CA, USA). After hybridization for 18 hours, extension, staining, and washing were performed successively. Finally, the BeadChip was imaged using the iScan system (Illumina, Inc.). In total, over 485,000 methylation sites were analyzed per sample, and the entire assay was performed according to the manufacturer’s instructions.

### Data processing

The raw files were processed by quality control and normalization using the software Genome Studio (Illumina). Then the methylation values of the tested sites were obtained as β-values, which were calculated as the methylation signal intensity divided by the sum of both methylation and unmethylation signals with background subtraction. β-values range from 0 to 1, representing completely unmethylated and fully methylated sites, respectively. Furthermore, sites with a detection p-value > 0.05 and sites containing missing values across all 72 samples were excluded.

### Statistical analysis of methylation level of functional regions

The Illumina Methylation Analyzer (IMA) package in the R language was used to calculate the methylation level of each of the following functional regions: TSS1500 (-1500 bp from the nearest TSS), TSS200 (-200 bp from the nearest TSS), 5’UTR, EXON1 (1^st^ exon of genes), 3’UTR, Gene Body, CpG islands, NSHORE (-2 kb region flanking the CpG island), SSHORE (+2 kb region flanking the CpG island), NSHELF (-4 to -2 kb region flanking the CpG island), and SSHELF (+2 to +4 kb region flanking the CpG island). The methylation level of each region was represented by the mean methylation level of all tested sites within each region.

### Software for data visualization

Hierarchical clustering was performed in R using the “heatmap” package. Moreover, the methylation pattern of the Y-chromosome was loaded into IGV for visualization.

## Results

### Analysis of haplogroups, sample selection, and preparation

**Fig 1:**
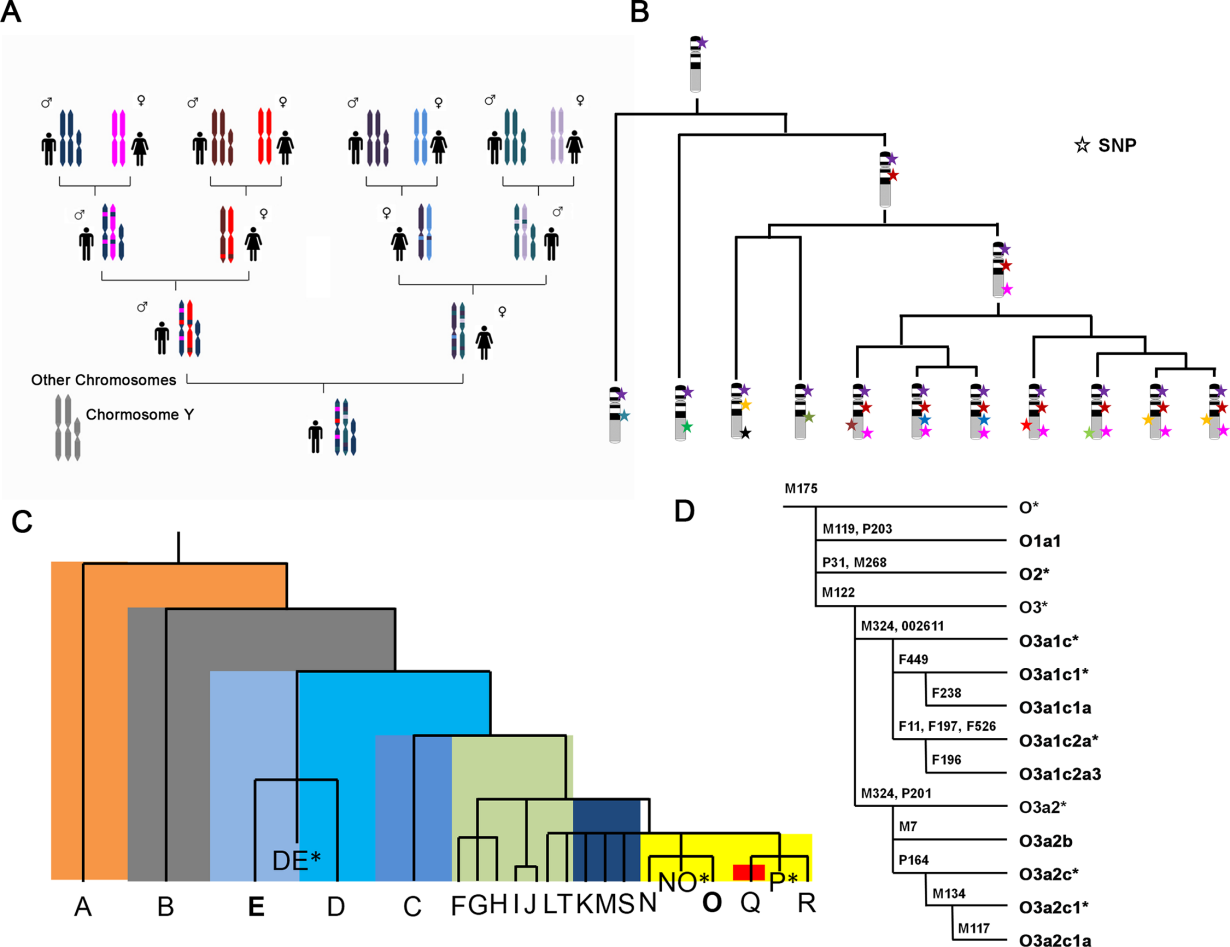
Characteristics of the Y chromosome and the Y chromosome phylogenetic tree. A) The abridged general view of stable inheritance of the Y chromosome among male generations. Recombination between homologous chromosomes are existed in other chromosomes. B) Illustration diagram of the SNP inheritance on the Y chromosome. Each color star represents a new SNP, and it can be inherited to the next generation. C) The abbreviated form of the Y chromosome phylogenetic tree. Haplogroup E and O were selected for this study. D) The tree of haplogroup O and its subgroups in Han Chinese population. The methylation patterns of bold labeled subclades are studied.

The inheritance of the Y chromosome is more stable than other chromosomes (Fig 1A). Deep-rooting pedigrees have been utilized to estimate the mutation rate of the Y chromosome specific microsatellites or base substitutions (Fig 1B) [42, 43]. Moreover, with single nucleotide polymorphism (SNP) analysis of the human Y-chromosome, researchers successfully found 13 haplotypes in a Japanese population [44]. We established several haplogroups with 72 samples using single nucleotide primer extension and SNP analysis based on the same method (Fig 1C and 1D).

Further, the DNA methylation level of all samples was detected using the Illumina 450K methylation microarray (Illumina, Inc., San Diego, CA, USA). We set up to determine whether the human epigenome was conserved by comparing the DNA methylation level of each haplogroup or subgroup. In this study, we focused on analyzing the methylation level of the Y chromosome. We tested the DNA methylation level of two technical replicates of two samples in advance. For all sites on the Y chromosome, the correlation of methylation profiles between two technical replicates was high (r > 0.99) (S1 Fig), indicating that this technology was robust for detecting the methylation pattern of the Y chromosome.

### DNA methylation pattern on the Y chromosome was similar in three families of haplogroup O2*

**Fig 2:**
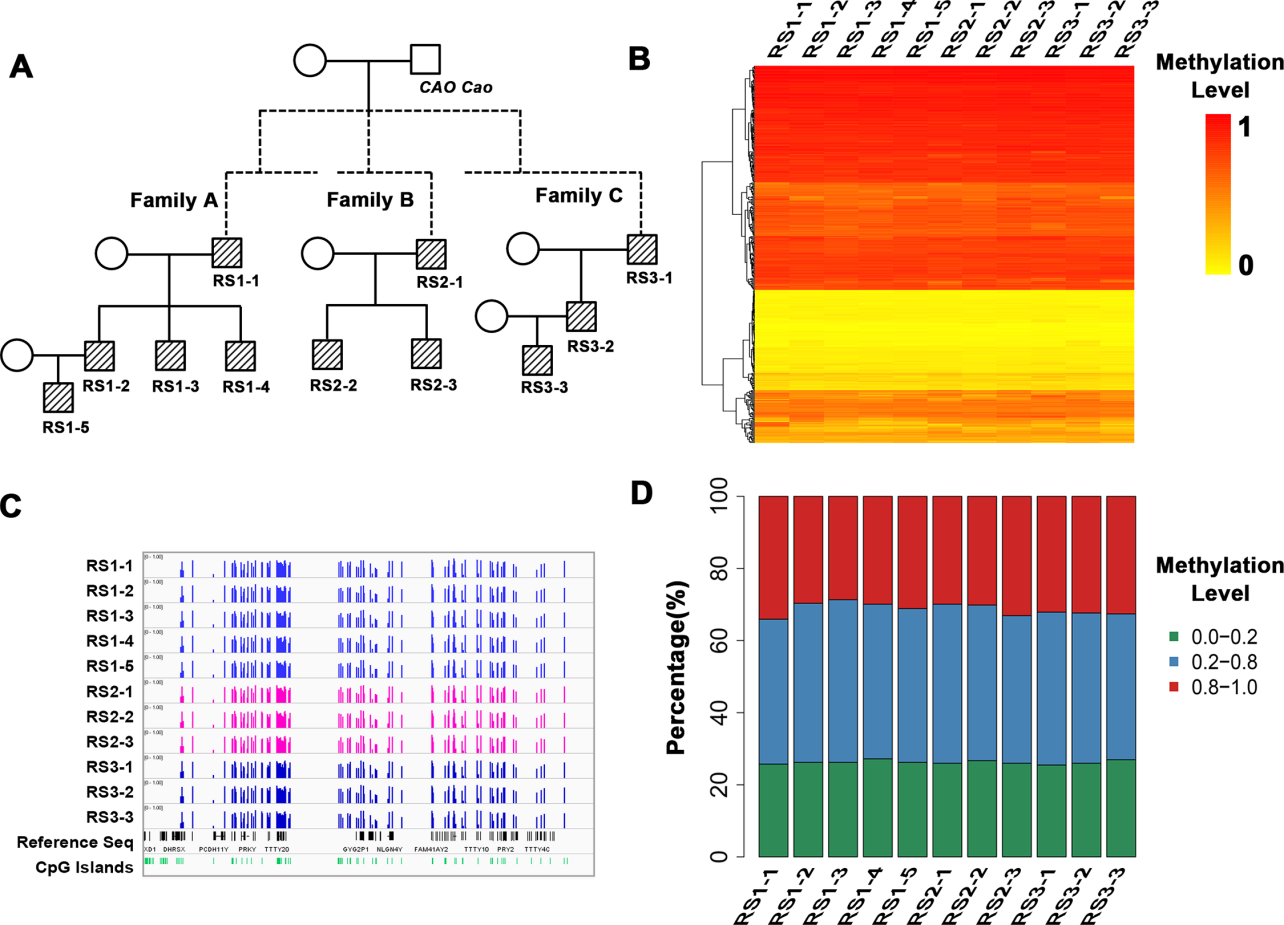
The conservative DNA methylation pattern on the Y chromosome within three haplogroup O2* families. A) Pedigree showing the relationship of family members. B) Heat map showing the methylation level of the Y chromosome in each sample. Each row line represents a single tested site, with each vertical line showing the β-value obtained in each individual sample. DNA methylation level is colored from orange to red to indicate low to high. C) The DNA methylation level is shown around the annotated genes and CpG Islands of the Y chromosome. D) Bar graphs showing the distribution of hyper-, intermediate-, and hypo-methylation sites on the Y chromosome in each sample respectively. Red indicates the hyper-methylation sites (β-value > 0.8), blue indicates intermediate-methylation sites (β-value: 0.2 to 0.8), and green indicates hypo-methylation sites (β-value < 0.2).

First, we selected 11 samples from Rushan, Shandong, China. They came from three families (families A, B, and C). They were all the descendants of Emperor CAO Cao (155AD–220AD), one of the most famous Emperors in China, according to the deep-rooting pedigrees [33, 34]. As shown in Fig 2A, two trios were included. They all belonged to haplogroup O2*, based on the Y chromosome haplogroup analysis (Fig 2A, S1 Table) [34].

404 sites on the Y chromosome were obtained for further analysis after filtering 12 CpG sites containing missing values or with detection p-values greater than 0.05. The data showed that there was no remarkable variation of the methylation pattern on the Y chromosome among family members or among different families (Fig 2B and 2C). Moreover, the distribution of hyper-methylation sites (β-value > 0.8), intermediate-methylation sites (β-value: 0.2–0.8), and hypo-methylation sites (β-value < 0.2) on the Y chromosome were similar for all individuals (Fig 2D). Specifically, the hyper-methylation sites and hypo-methylation sites were highly consistent within family members (S2 Fig).

To further investigate whether methylation pattern on the Y chromosome is correlated with relations, the mean β-value of each site was calculated. We did not find any family A-specific methylation sites (Wilcoxon Rank-sum Test, FDR-adjusted p < 0.05, and |beta_difference| ≥ 0.2) by comparing the mean β-value of family A with the mean β-value of the other two families (family B and family C). There was also no family-specific methylation pattern between family B and family C.

### DNA methylation pattern on the Y chromosome was conservative among different geographic subgroups of haplogroup O2*

**Fig 3:**
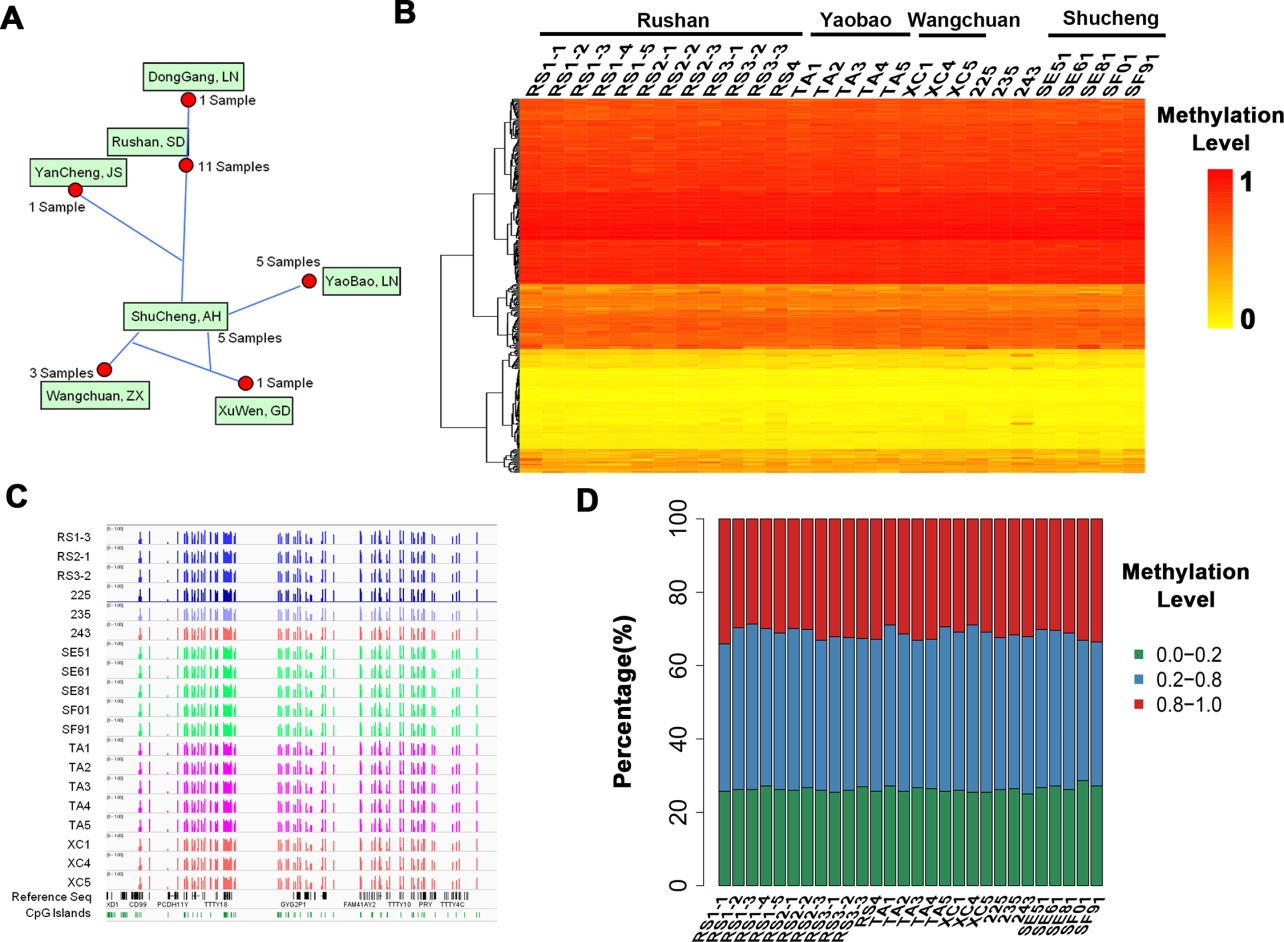
The similar DNA methylation pattern on the Y chromosome among different geographic subgroups of haplogroup O2*. A) Geographic distribution of 28 haplogroup O2* individuals. B) Heat map showing the methylation level of the Y chromosome in each sample. Each row line represents a single site, with each vertical line showing the β-value obtained in each individual sample. DNA methylation level is colored from orange to red to indicate low to high. C) The DNA methylation level is shown around the annotated genes and CpG Islands of the Y chromosome. D) Bar graphs showing the distribution of hyper-, intermediate-, and hypo-methylation sites on the Y chromosome in each haplogroup O2* sample.

Given the above results, we speculated whether the methylation level of the Y chromosome was also conserved among subgroups of haplogroup O2*. To test this hypothesis, whole blood samples were collected from 17 healthy male individuals of haplogroup O2* from different regions (Donggang, Yancheng, Shucheng, Yaopu, Wangchuan, and Xuwen) (Fig 3A, S1 Table) [34]. The result showed that there hardly no variation among geographic groups regarding the overall methylation pattern (Fig 3B and 3C). The distribution of hyper-methylation, intermediate-methylation, and hypo-methylation sites were similar among all individuals (Fig 3D). Further, no any geography-specific methylation sites were found in haplogroup O2* (Wilcoxon Rank-sum Test, FDR-adjusted p < 0.05, and |beta_difference| ≥ 0.2).

The common hyper-methylation sites (mean β-value > 0.8) and hypo-methylation sites (mean β-value < 0.2) among all above samples were selected for further investigation. The values of standard deviation (SD) of the common hyper-methylation sites were less than 0.1 (S3A Fig), indicating that DNA methylation pattern was relatively conserved. However, there was a few variation of DNA methylation in the hypo-methylation sites (the values of SD in several sites were larger than 0.1) (S3A Fig). Further, we assessed the methylation level of these common hyper- and hypo-methylation sites of each sample. We found that the methylation level of common hyper-methylation sites were similar among all O2* samples, showing no correlation with the family relation or geographic factors (S3B Fig). However, we found quite a few remarkably different methylation sites in Sample 243 within the common hypo-methylation sites (S3C Fig). Because we analyzed a total of 28 samples from haplogroup O2*, the variation of Sample 243 was not haplogroup-specific. One possibility was that the differential methylation pattern of Sample 243 was individualized unique.

All these results suggested that the DNA methylation pattern on the Y chromosome was conserved within haplogroup O2* samples.

### Specific methylation sites were found in haplogroup O3a2b samples

**Fig 4:**
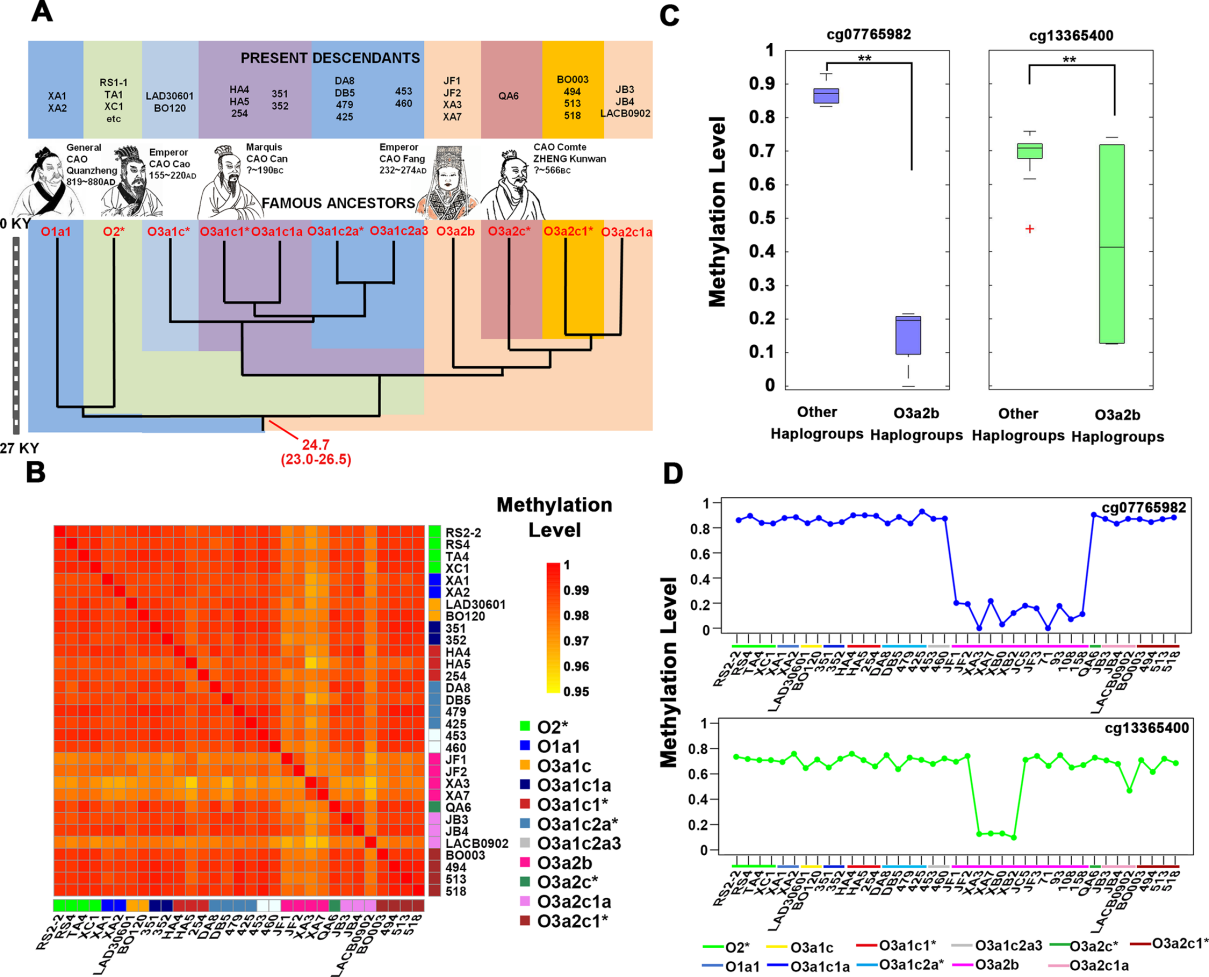
Haplogroup-specific DNA methylation variation in haplogroup O3a2b samples. A) The information of Y haplotype and coalescence time for samples. The left bar graph indicates the coalescence time, from 27 thousand years ago (kya) to now. Numbers in red indicate the coalescence time (in years) and 95% confidence interval of the node. B) Heat map showing the Pearson correlation coefficients among different haplogroups. Correlation coefficients are colored yellow to red to indicate low to high, respectively. C) Box plots showing the haplogroup O3a2b-specific methylation sites. **P < 0.01. D) The methylation level of cg07765982 and cg13365400 within all samples. Each data point represents the β-value of each sample.

We collected whole blood samples from 27 healthy male donors to analyze their DNA methylation patterns on the Y chromosome according to the most recent Y chromosome phylogenetic tree. These samples came from different haplogroups (O1a1-P203, O3a1c*-002611, O3a1c1*-F449, O3a1c1a-F238, O3a1c2a*-F11/F197/F526, O3a1c2a3-F196, O3a2b-M7, O3a2c*-P164, O3a2c1*-M134, and O3a2c1a-M117) (Fig 4A, S1 Table) [41, 45]. Sample RS2-1, RS4, TA4, and XC1 were randomly selected to be representative of haplogroup O2*. The divergence time of these haplogroups was approximately 24.7 thousand years ago (kya) [39].

To determine whether the DNA methylation pattern on the Y chromosome was also conservative among different haplogroups, Pearson correlation coefficients between each haplogroup were calculated. The result showed that the correlation of most haplogroups was high (Fig 4B). However, relatively low correlation between haplogroup O3a2b (JF1, JF2, XA3, XA7) and other haplogroups was found. Moreover, the sample LACB0902 in haplogroup O3a2c1a also showed weak correlation with other samples (Fig 4B).

We found two specific methylation sites in haplogroup O3a2b samples by comparing mean β-values between different haplogroups (Target ID: cg07765982 and cg13365400) (Fig 4C). To confirm the existence of these two haplogroup-specific sites, we collected another 8 male samples of haplogroup O3a2b. These 8 samples were collected from 6 distant provinces of China to exclude the influence of geographic factor on the methylation pattern. Overall, all of 12 samples from haplogroup O3a2b shared one haplogroup-specific methylation site (cg07765982) (S4A Fig, S2 Table).

The methylation level of cg07765982 was low in all haplogroup O3a2b samples (Fig 4D). The analysis began with four haplogroup O3a2b samples (XA3, XA7, XB0, XB2) sharing the hypomethylation level at cg13365400 site (Fig 4D). These four samples were all collected from Shexian, Anhui, China (S4B Fig). Further, 2 other samples (XA1, XA2) that also came from Shexian, Anhui, China were included, which belonged to haplogroup O1a1 (O-P203). By comparing the methylation pattern of cg13365400 among the above 6 samples (one geographical position, two different haplogroups), we found that the difference was haplogroup-specific rather than geography-specific (S4C Fig). One explanation for the above data was that the methylation variation of cg07765982 existed in the common ancestor of haplogroup O3a2b and was inherited by all the descendants.

We further tested 4 additional haplogroup O3a2c1a samples to assess whether the methylation pattern of sample LACB0902 was haplogroup O3a2c1a-specfic. 8 variant sites were selected with a cut-off SD > 0.1 after calculating the standard deviation of the β-value of each site among all 7 haplogroup O3a2c1a samples. However, these sites were only differentially methylated in sample LACB0902 (S4D Fig), indicating that the difference of the methylation pattern in sample LACB0902 was individual rather than haplogroup-specific.

### Haplogroup O3a2b-specific methylation sites were located in gene body region on the Y chromosome

According to the human reference sequence (hg19), the tested sites on the Y chromosome were distributed on 11 regions: TSS1500 (-1500 bp from the nearest TSS), TSS200 (-200 bp from the nearest TSS), 5’UTR, EXON1 (1^st^ exon of genes), 3’UTR, Gene Body, CpG islands, NSHORE (-2 kb region flanking the CpG island), SSHORE (+2 kb region flanking the CpG island), NSHELF (-4 to -2 kb region flanking the CpG island), and SSHELF (+2 to +4 kb region flanking the CpG island) (S3 Table). The mean methylation level of all tested sites within each region was taken as this region’s methylation index.

**Fig 5:**
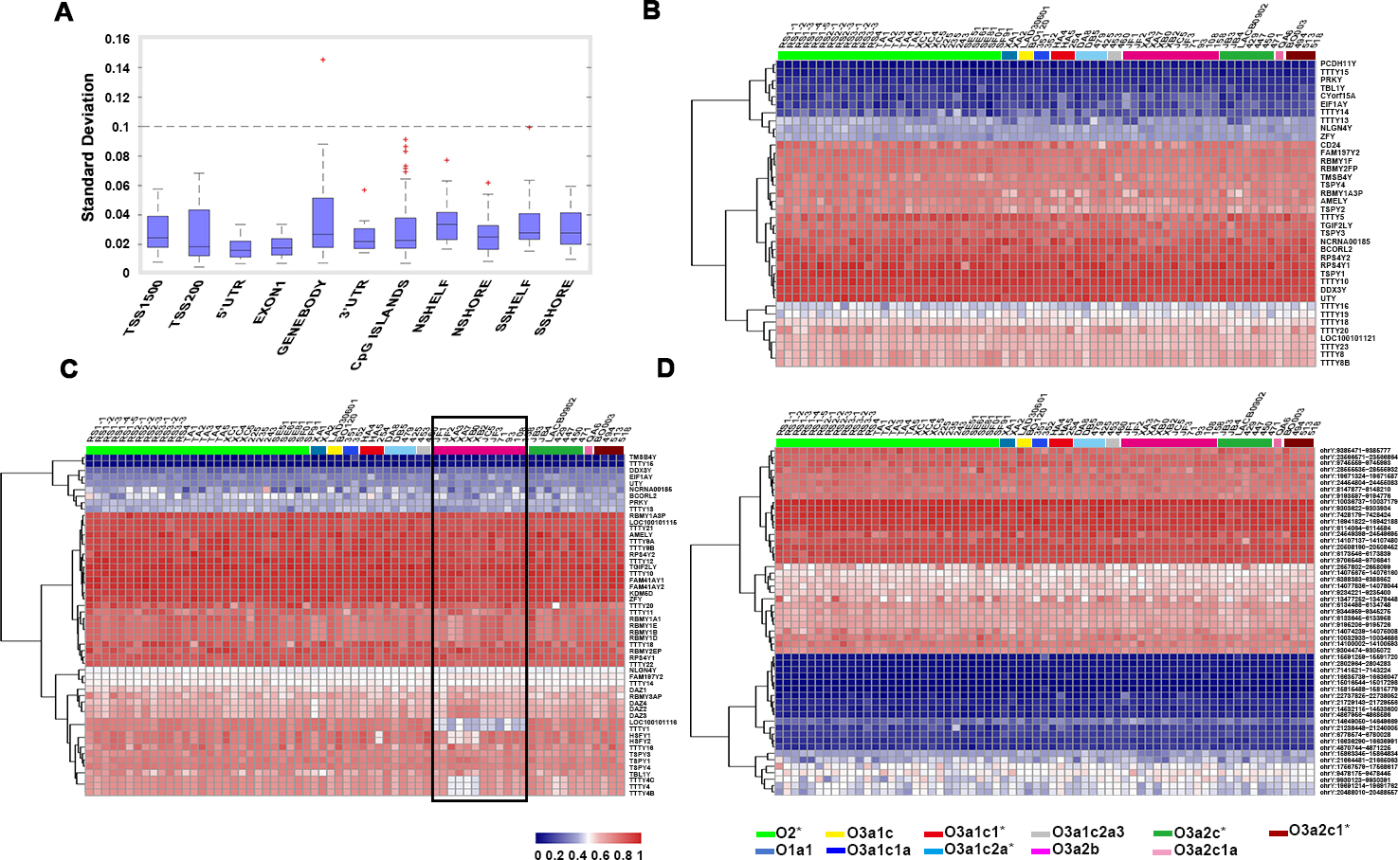
The methylation pattern of functional regions on the Y chromosome. A) Box plots showing the standard deviation of methylation level within each region. The median line indicates the average methylation level, the edges represent the 25th/75th percentile, and the whiskers represent the 2.5th/97.5th percentile. B−D). Heat map showing the methylation levels of 38 detected TSS1500 regions (B), 53 gene body regions (C), and 55 CpG island regions (D).

We found that the variation in gene body region was greater than in other regions by calculating the standard deviation of each region among all samples (Fig 5A). Further, we assessed the overall methylation pattern of 53 tested genes. Result showed that the methylation pattern of two genes was haplogroup O3a2b-specific (*LOC100101116*, *TTTY1*) (Fig 5C). However, we did not find such a haplogroup-specific variation on the other 10 functional regions (Fig 5B and 5D, S5 Fig).

### One haplogroup E-specific methylation site was found with a remote divergence time

**Fig 6:**
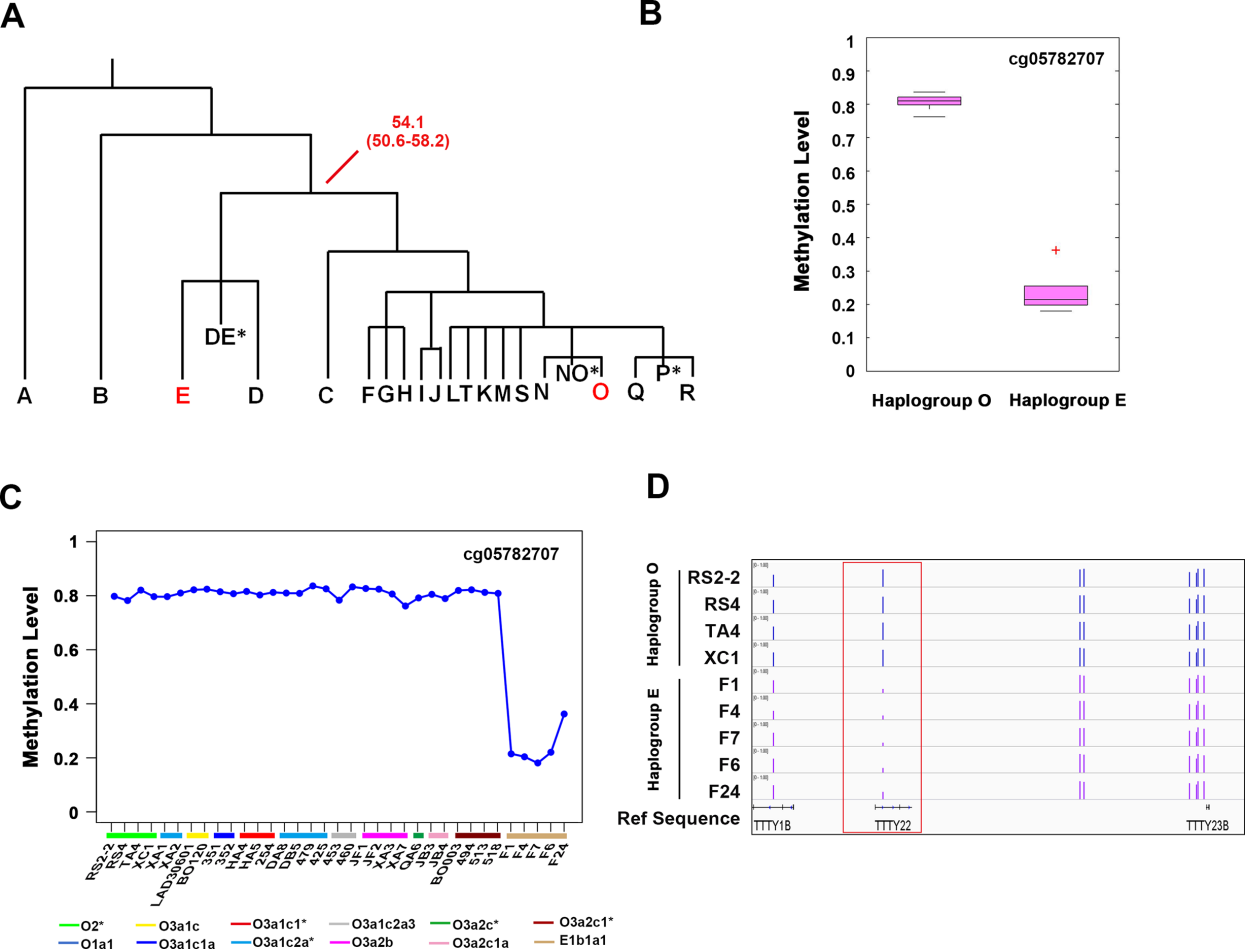
The different methylation site in haplogroup E1b1a1 samples. A) The Y chromosome phylogenetic tree showing the coalescence time between haplogroup E and haplogroup O. Numbers in red indicate the coalescence time (in years) and 95% confidence interval of the node. B) Box plots showing the methylation level of haplogroup E1b1a1-specific methylation site. **P < 0.01. C) The methylation level of cg05782707 among all samples. Each data point represents the β-value of each sample. D) The genomic location of cg05782707 site.

To further test our theory, we collected 5 samples from Nigeria, Africa. These male donors came from haplogroup E1b1a1 (E-M2), and they had diverged from haplogroup O over 54.1 thousand years ago (Fig 6A). After filtering sites with detection p-values > 0.05 and with missing values, the remaining sites on the Y chromosome were used for further investigation.

As shown in Fig 6B, we found one haplogroup E1b1a1-specific methylation site (cg05782707) (Fig 6B, S4 Table), which was relatively hypo-methylated in all 5 haplogroup E samples (Fig 6C). We found that this site located in the gene body region of *TTTY22* after mapping it to the human reference sequence (hg19) (Fig 6D).

### The haplogroup-specific methylation sites identified on the Y chromosome were genotype-dependent

Quite a few studies revealed that DNA mutations adjacent to the CpG site can affect its methylation level [46, 47]. To determine whether the haplogroup O3a2b-specific methylation site was affected by SNPs, we performed mutation detection analysis. Result suggested that the haplogroup O3a2b-specific methylation was accompanied with genetic mutation at the corresponding CpG site (S6 Fig). We further found that the genomic deletion around haplogroup E-specific methylation site contributed to its specific methylation level. Taken together, the above two haplogroup-specific methylation sites were both genotype-dependent.

### DNA methylation on other chromosomes were not as conserved as on the Y chromosome

Further, we selected the other two chromosomes to analyze their variance among all samples. We found that the degree of variation on the other two chromosomes was greater than that on the Y chromosome by assessing the standard deviation of each detected site across all samples (S7A Fig). We performed principal component analysis and found that the methylation pattern on the other two chromosomes were variable within the same haplogroup using chromosome 12 and the X chromosome as examples (S7B Fig). Therefore, the DNA methylation pattern on other chromosomes was not conserved during human history. This complicated pattern maybe caused by frequent spontaneous recombination between sister chromatids [48–50]. Further, we found there were family-specific methylation variations through investigating the methylation pattern on all chromosomes between 3 families of haplogroup O2* (S8A Fig). Moreover, we found more haplogroup-specific methylation sites between haplogroup O2* and haplogroup O3 (S8B Fig).

**Fig 7:**
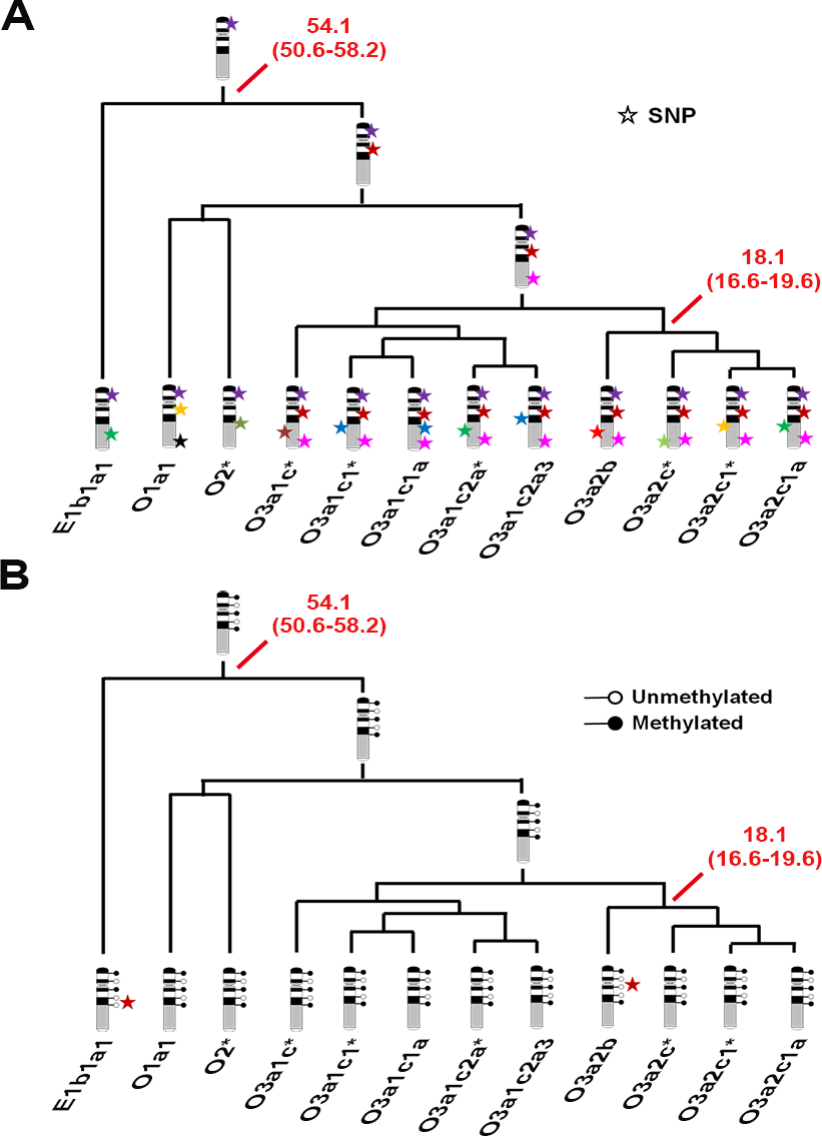
The inheritance schematic model of the DNA methylation pattern on the Y chromosome. A) Illustration model of the SNP inheritance on the Y chromosome. Each color star represents a new SNP, and it can be inherited to the next generation. B) The inheritance schematic model of DNA methylation pattern on the Y chromosome. Red star indicates a haplogroup-specific methylation site. This model shows the stable methylation pattern on the Y chromosome within each haplogroup. White circles represent unmethylated sites, black circles represent the methylated sites. Numbers in red indicate the coalescence time (in years) and the 95% confidence interval of the node.

It is well known that SNPs can be inherited and accumulated during human male evolution (Fig 7A). In this study, we also found that the methylation pattern on the Y chromosome could also be stably inherited during human male history (Fig 7B). Our result demonstrated an interesting fact that the DNA methylation pattern on the Y chromosome was relatively stable during evolution.

## Discussion

It is well acknowledged that Y chromosome is a powerful tool for human evolution study, since it is transferred between males and remains relative stability after multi-generation inheritance. Based on this powerful tool, we analyzed a pedigree from haplogroup O2*. By analyzing their DNA methylation level, we found that the DNA methylation pattern on the Y chromosome was conserved within haplogroup O2*, even though the divergence time between these samples was approximately 1800 years ago. Further, we investigated the DNA methylation pattern among different haplogroups. We detected two haplogroup O3a2b-specific methylation sites (cg07765982 and cg13365400). The variant methylation site cg13365400 existed in 4 samples (Shexian, Anhui, China), and this variation was haplogroup-specific rather than geography-specific. Interestingly, after performing DNA mutation analysis, we found that these sites were accompanied by SNPs. Moreover, we found a haplogroup E-specific methylation site (cg05782707) with a remote divergence time, and this site was also associated with genetic mutation.

Several studies showed that the genome-wide DNA methylation underwent methylation reprogramming during early embryonic development [17–20]. In particular, the methylation of CpG Islands on the Y chromosome also underwent the above process (S9 Fig). However, one key question in this field is whether epigenetic modification, such as DNA methylation, can be stably passed from generation to generation like the DNA sequence. In our study, based on deep-rooted pedigrees and the Y chromosome phylogenetic tree, we found that the methylation pattern on the Y chromosome could also be stably inherited during human male history.

It is well known that Y chromosome had lost more than 1,393 genes since its existence, giving a rate of genetic loss of 4.6 genes per million years [51]. However, human Y chromosome had not lost any genes since humans had diverged from chimpanzees, dating back to 6–7 million years ago [52]. It indicates that gens on the Y chromosome have important roles in human development. Our work indicated that the basic DNA methylation pattern on the Y chromosome could be stably passed from generation to generation like the DNA sequence for almost 50 thousand years, indicating that DNA methylation may also play an important role in the development and evolution of human males.

Interestingly, we also found that all haplogroup-specific DNA methylation variations were genetically related. One possible explanation is that although basic DNA methylation pattern is stable, environmentally caused DNA methylation variation could not be inherited between generations unless it is genetically SNPs related. The fact that these individual variations of the DNA methylation could not be passed between generations may partially be resulted from DNA methylation reprogramming, which erases and resets whole genome DNA methylation during development.

Our study was conducted with DNA from peripheral blood cells, which do not express Y chromosome genes in any significant manners. Accordingly, the relative stable DNA methylation pattern on the Y chromosome in other tissues or cells will need further study. Additionally, Daniel *et al*. found that the surviving genes with X homologues on the Y chromosome were dosage-sensitive, which function as regulators of transcription, translation and protein stability [27]. Further, we detected the DNA methylation pattern of promoter regions of these genes to study the potential effects of DNA methylation on these genes. Results showed that the methylation level of these genes was conserved among different haplogroup samples. Meanwhile, most of these genes were hyper-methylated within their promoter regions, indicating their relatively inactive state in peripheral blood cells.

The DNA methylation on the Y chromosome is relatively stable. This indicates that the Y chromosome is not as fragile as previously suggested [51, 53–56]. The function and phenotypes of human males have been protected by a stable DNA methylation pattern for tens of thousands of years. Our work also provided a unique research platform to study epigenetic-related diseases in the future. With the aid of deep-rooted pedigrees and the Y chromosome phylogenetic tree, we can perform novel family pedigree associated epigenetic studies for certain diseases.

## Supporting Information

S1 Fig Scatterplots showing Pearson correlation coefficients between two repeated analyses of two samples. (TIFF)

S2 Fig The common hyper- and hypo-methylation sites among each family samples. A) Venn diagram showing the overlap of hyper-methylation sites among samples of family A, family B, and family C, respectively. B) Venn diagram showing the overlap of hypo-methylation sites among samples of family A, family B, and family C, respectively.
(TIFF)

S3 Fig The DNA methylation level of the Y chromosome within haplogroup O2* samples. A) Box plots showing the distribution of standard deviation among the methylation levels of common hyper- and hypo-methylation sites in haplogroup O2* samples. B) Box plots illustrating the methylation level of common hyper-methylation sites in each sample. C) Box plots illustrating the methylation level of common hypo-methylation sites in each sample. The median line indicates the average methylation level, the edges represent the 25th/75th percentile, and the whiskers represent the 2.5th/97.5th percentile.
(TIFF)

S4 Fig The DNA methylation pattern on the Y chromosome among different haplogroup samples. A) The methylation level of cg07765982 and cg13365400 between different haplogroups. **P < 0.01. B) The methylation level of cg13365400 within all 12 haplogroup O3a2b samples. C) The methylation level of cg13365400 within 6 samples (one geographical position, two different haplogroups). D) DNA methylation level of 8 LACB0902 unique methylation sites in all haplogroup O3a2c1a samples. Each data point represents the β-value obtained in each sample.
(TIFF)

S5 Fig The methylation pattern of another 8 functional regions. A−G). Heat map showing the average methylation levels of TSS200 region (A), 5’UTR region (B), EXON1 region (C), 3’UTR region (D), NSHELF region (E), NSHORE region (F), SHELF region (G) and SHORE region (H).
(TIFF)

S6 Fig The genotype analysis of the haplogroup O3a2b-specific methylation site. Sanger sequencing showing a nucleotide mutation within the haplogroup O3a2b samples.
(TIFF)

S7 Fig Stable DNA methylation pattern on the Y chromosome. A) Box plots showing the distribution of standard deviation of the methylation levels on each chromosome. The median line indicates the average methylation level, the edges represent the 25th/75th percentile, and the whiskers represent the 2.5th/97.5th percentile. B) Principal component analysis of the methylation pattern on chromosome 12, the X chromosome, and the Y chromosome in all samples. Each data point represents an individual sample.
(TIFF)

S8 Fig Whole genome DNA methylation analysis of three haplogroup O2* families and different haplogroups. A) Heat map showing the family-specific DNA methylation sites on whole genome. B) Heat map showing the haplogroup O2* and haplogroup O3-specific DNA methylation sites on whole genome. Each vertical line represents a single site, with each row showing the β-value obtained in each individual tested.
(TIFF)

S9 Fig The DNA methylation reprogramming process during early human embryonic development. Published methylation data showing a de-methylation and then re-methylation process during early human embryonic development. Each data point represents the mean β-value of each stage.
(TIFF)

S1 Table Sample information. (TIFF)

S2 Table Haplogroup O3a2b-specific methylation site. (TIFF)

S3 Table Eleven regional categories on the Y chromosome. (TIFF)

S4 Table Haplogroup E1b1a1-specific methylation site. (TIFF)
