# Supplementary figures and images for: Epigenetic Pattern on the Human Y Chromosome Is Evolutionarily Conserved
Source: PLoS One. 2016 Jan 13;11(1):e0146402. doi: 10.1371/journal.pone.0146402 (PMC4711989; doi:10.1371/journal.pone.0146402)

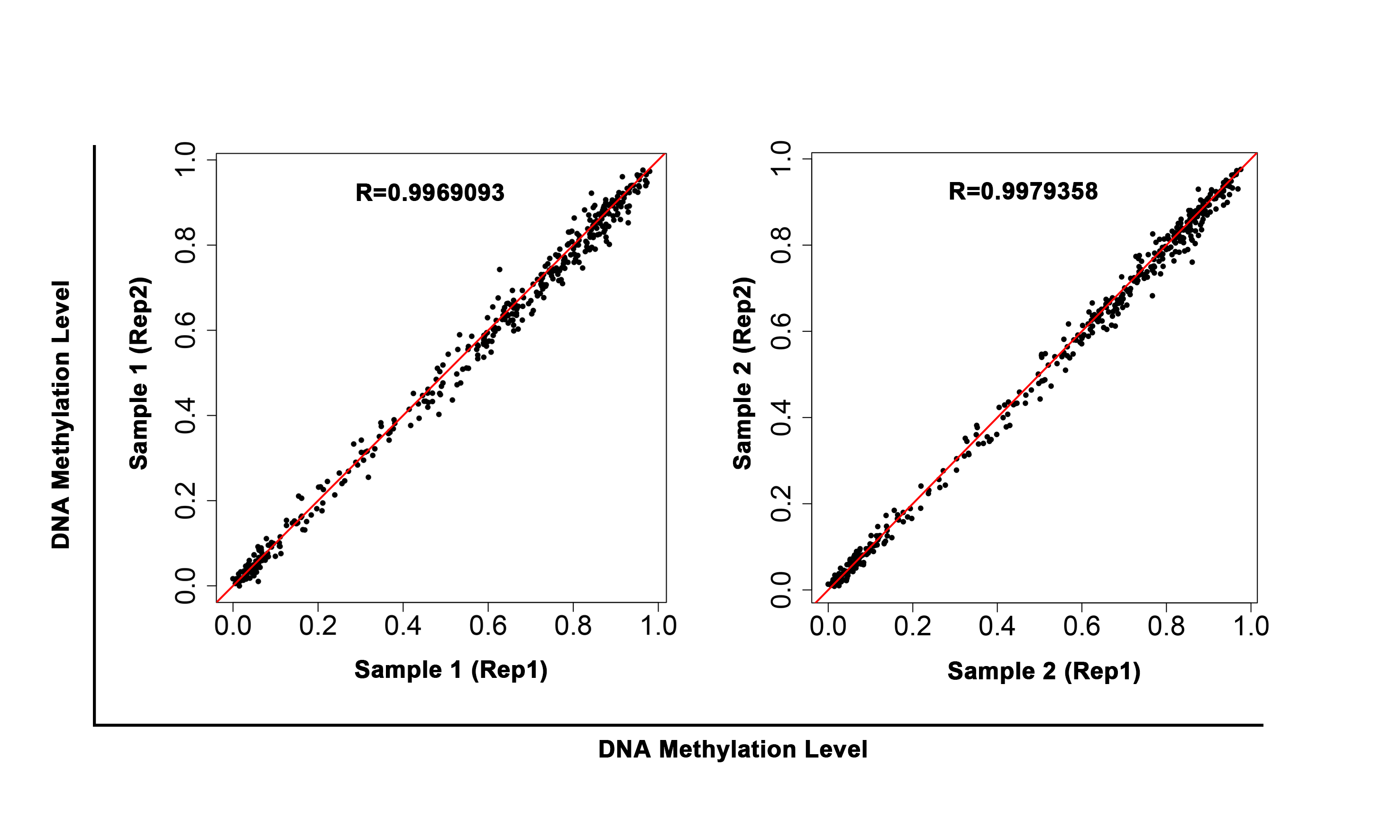

Supplement: S1 Fig — (TIFF) [file pone.0146402.s001.tiff]

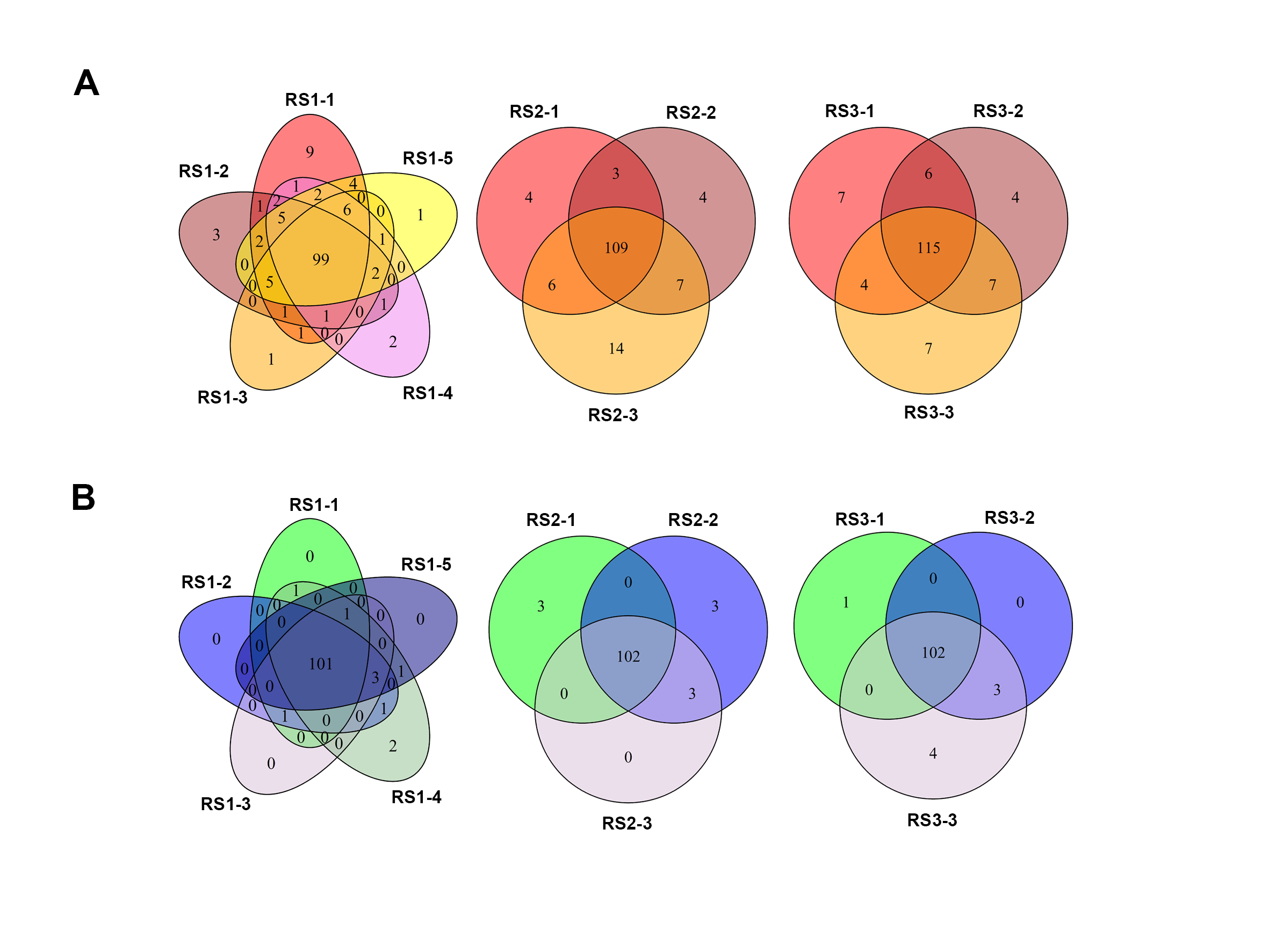

Supplement: S2 Fig — A) Venn diagram showing the overlap of hyper-methylation sites among samples of family A, family B, and family C, respectively. B) Venn diagram showing the overlap of hypo-methylation sites among samples of family A, family B, and family C, respectively. (TIFF) [file pone.0146402.s002.tiff]

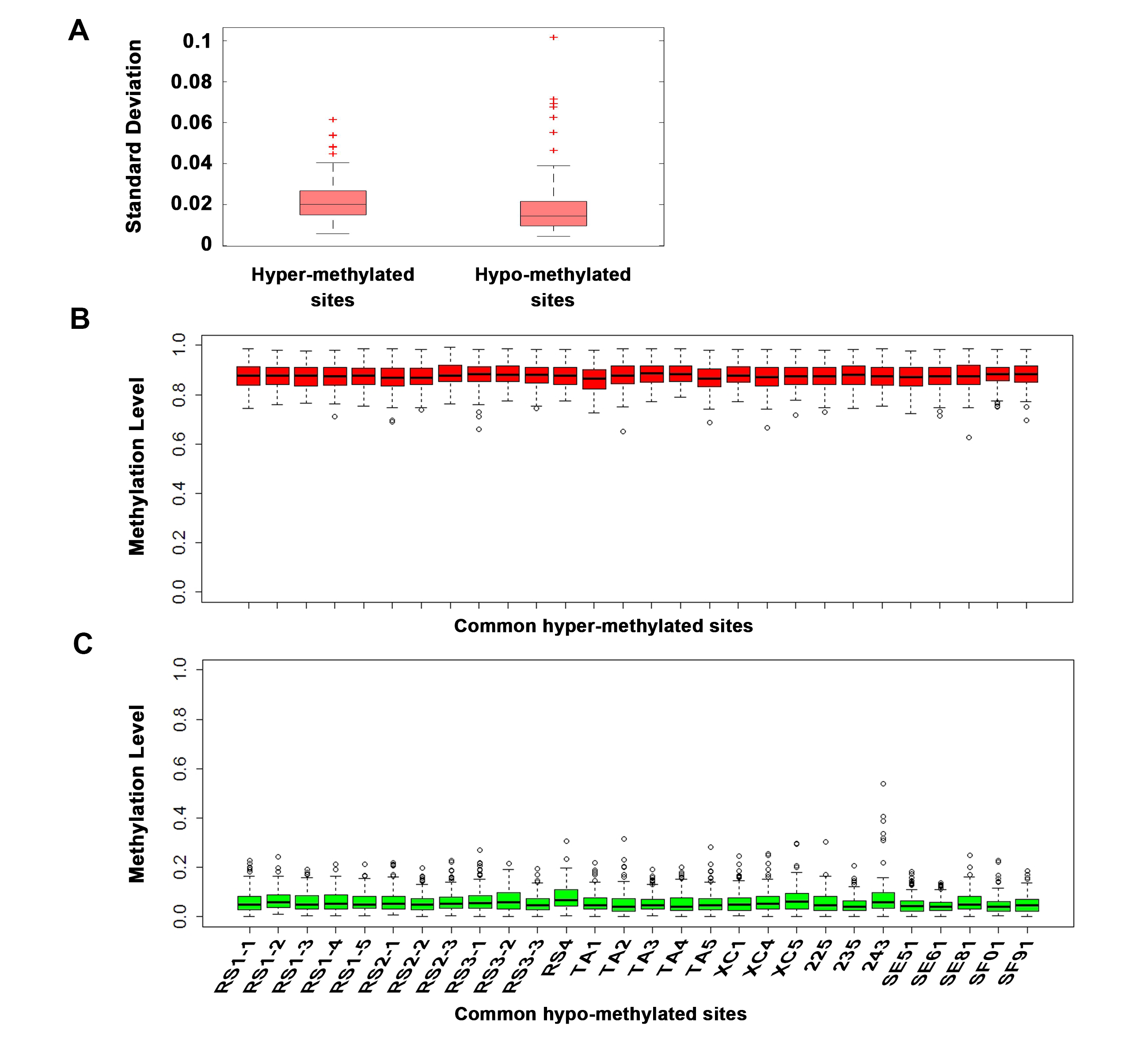

Supplement: S3 Fig — A) Box plots showing the distribution of standard deviation among the methylation levels of common hyper- and hypo-methylation sites in haplogroup O2* samples. B) Box plots illustrating the methylation level of common hyper-methylation sites in each sample. C) Box plots illustrating the methylation level of common hypo-methylation sites in each sample. The median line indicates the average methylation level, the edges represent the 25th/75th percentile, and the whiskers represent the 2.5th/97.5th percentile. (TIFF) [file pone.0146402.s003.tiff]

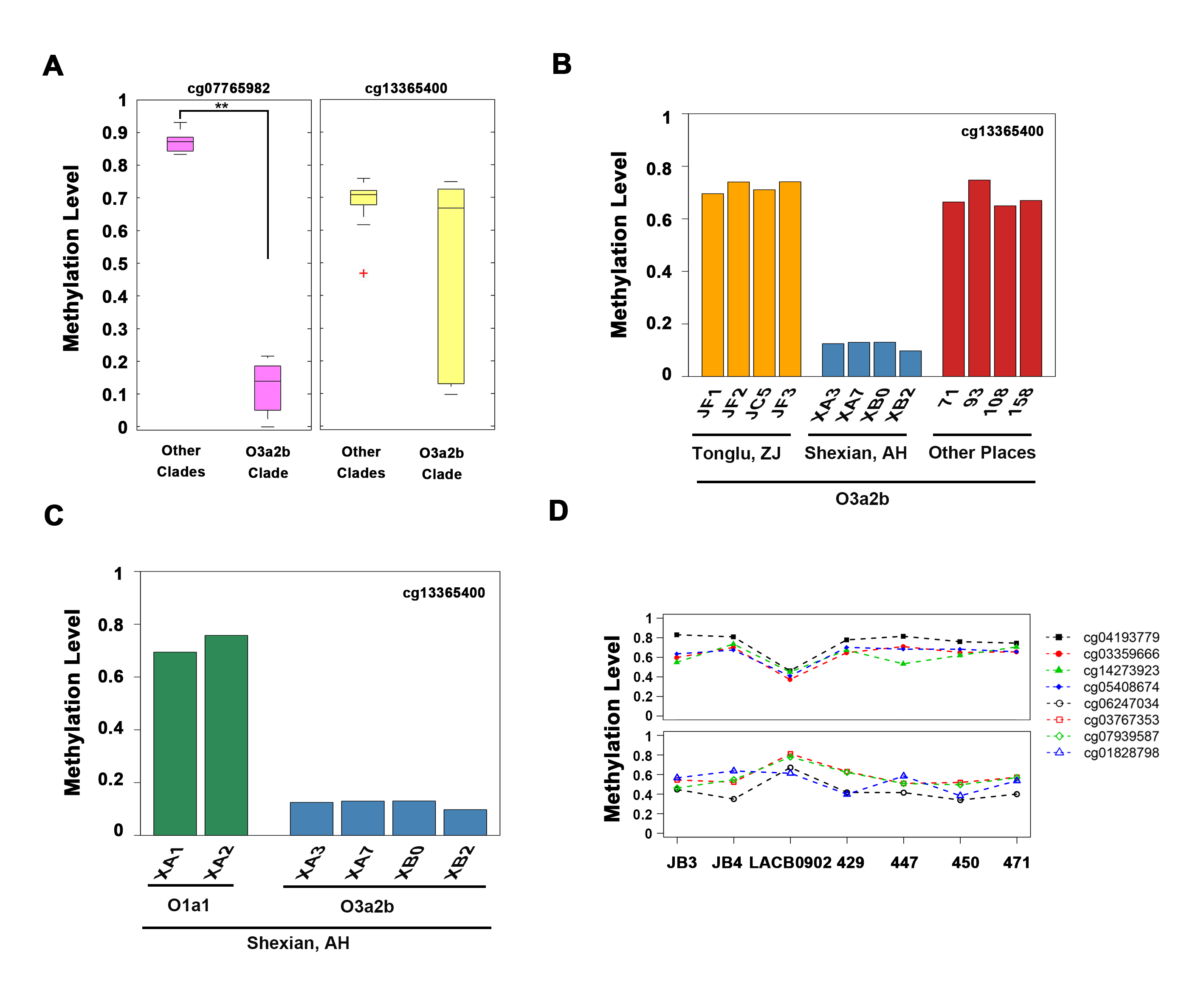

Supplement: S4 Fig — A) The methylation level of cg07765982 and cg13365400 between different haplogroups. **P < 0.01. B) The methylation level of cg13365400 within all 12 haplogroup O3a2b samples. C) The methylation level of cg13365400 within 6 samples (one geographical position, two different haplogroups). D) DNA methylation level of 8 LACB0902 unique methylation sites in all haplogroup O3a2c1a samples. Each data point represents the β-value obtained in each sample. (TIFF) [file pone.0146402.s004.tiff]

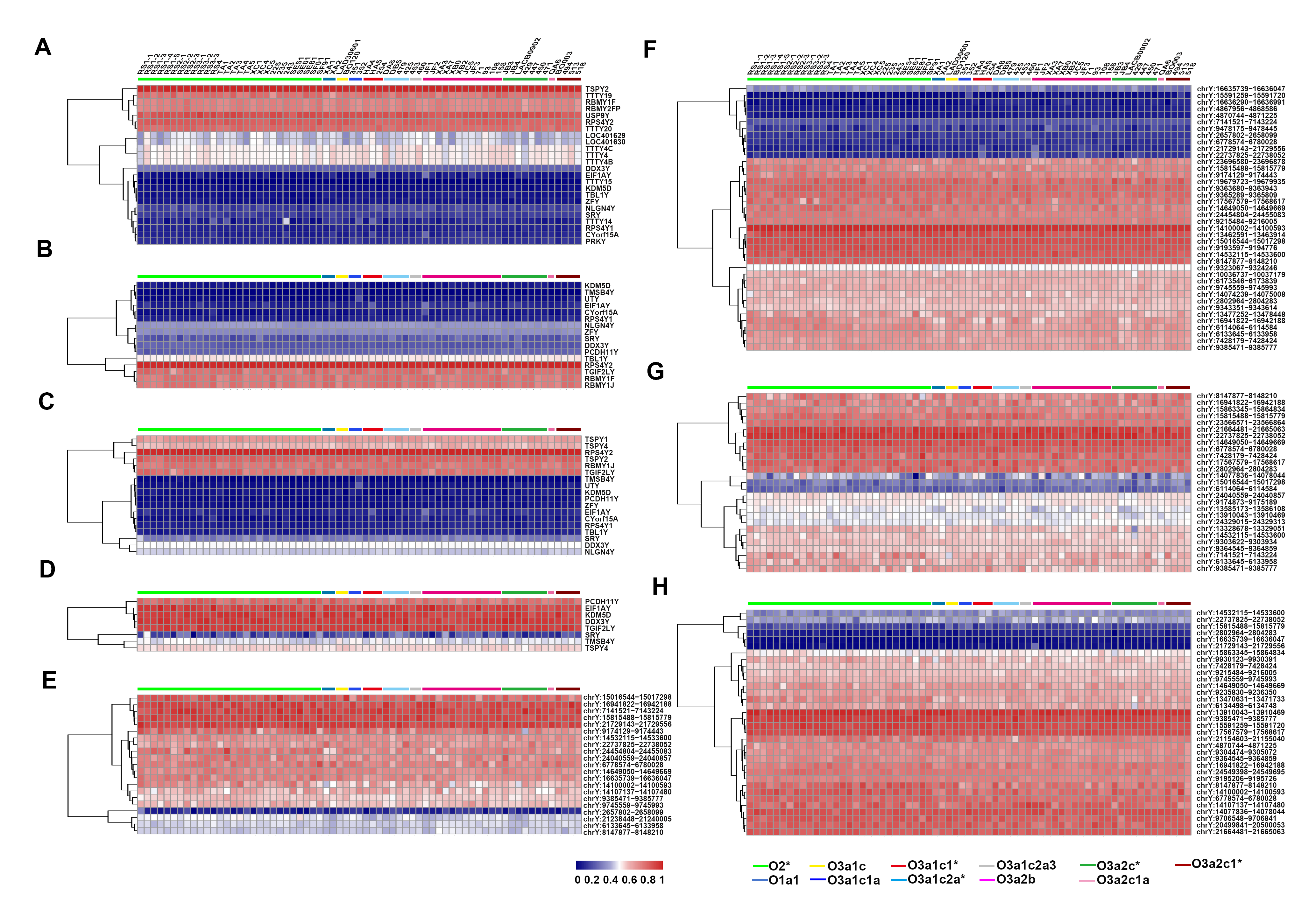

Supplement: S5 Fig — A−G). Heat map showing the average methylation levels of TSS200 region (A), 5’UTR region (B), EXON1 region (C), 3’UTR region (D), NSHELF region (E), NSHORE region (F), SHELF region (G) and SHORE region (H). (TIFF) [file pone.0146402.s005.tiff]

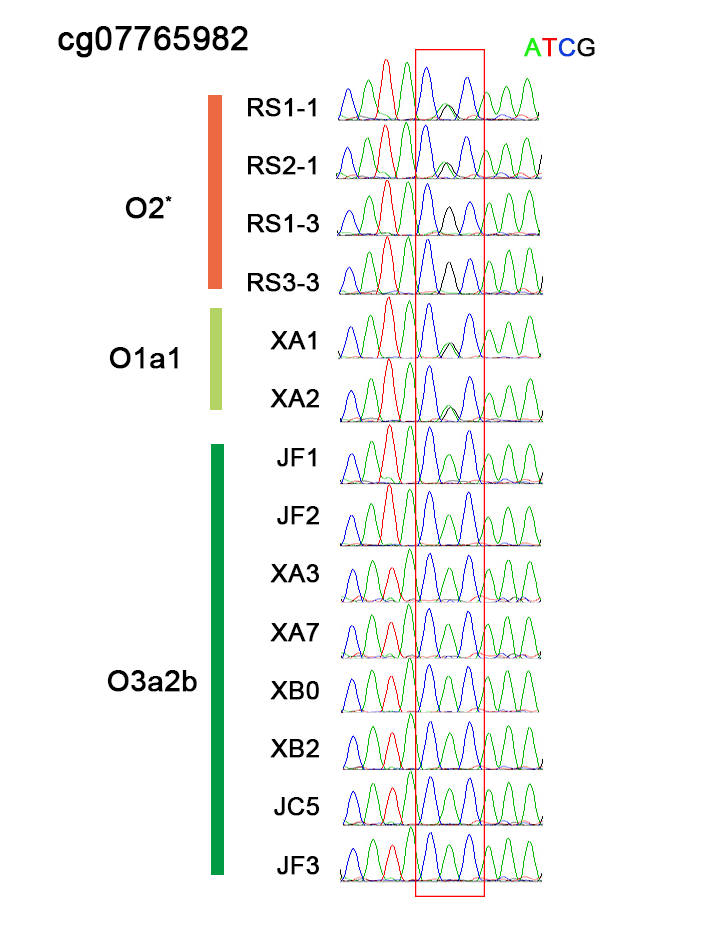

Supplement: S6 Fig — Sanger sequencing showing a nucleotide mutation within the haplogroup O3a2b samples. (TIFF) [file pone.0146402.s006.tiff]

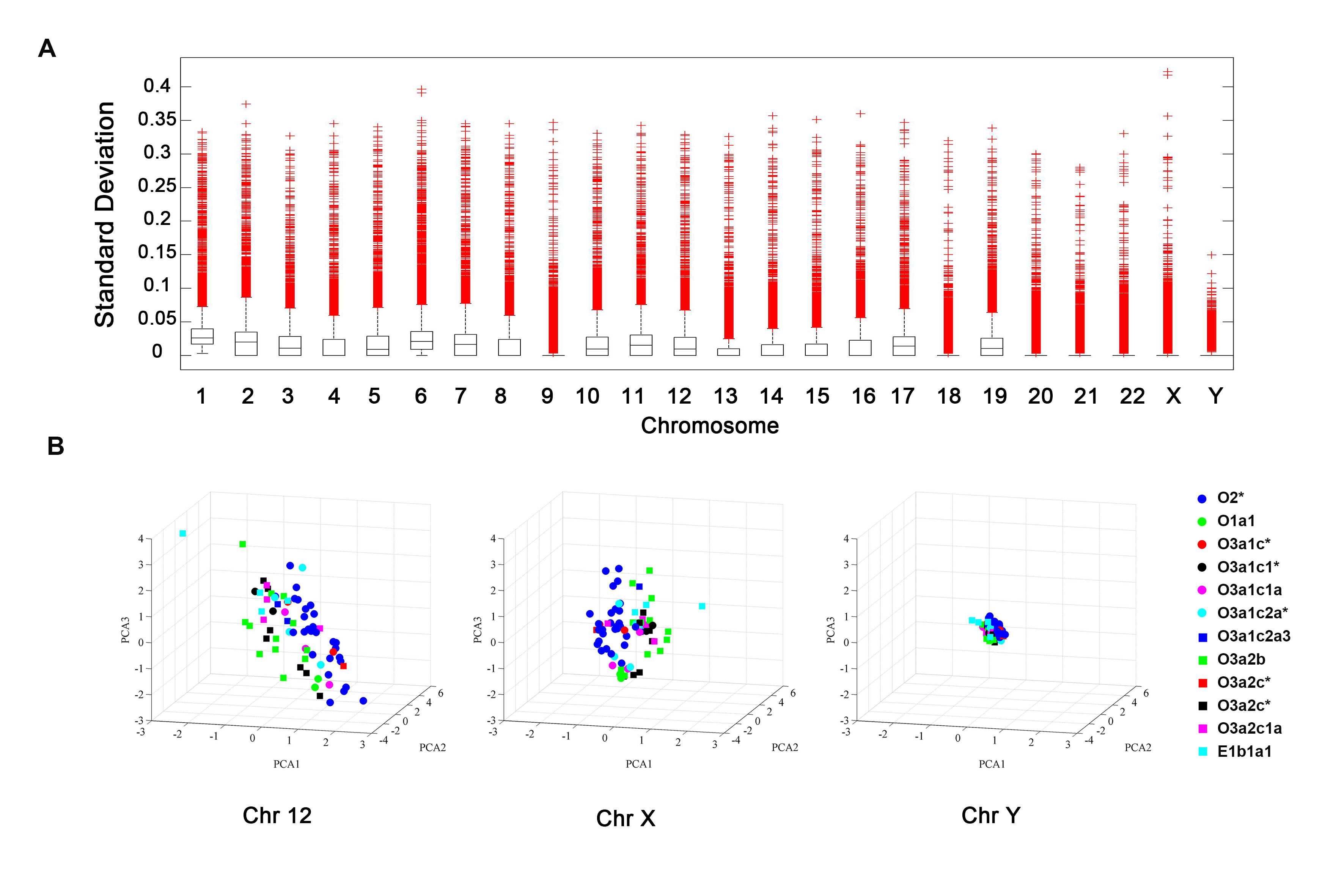

Supplement: S7 Fig — A) Box plots showing the distribution of standard deviation of the methylation levels on each chromosome. The median line indicates the average methylation level, the edges represent the 25th/75th percentile, and the whiskers represent the 2.5th/97.5th percentile. B) Principal component analysis of the methylation pattern on chromosome 12, the X chromosome, and the Y chromosome in all samples. Each data point represents an individual sample. (TIFF) [file pone.0146402.s007.tiff]

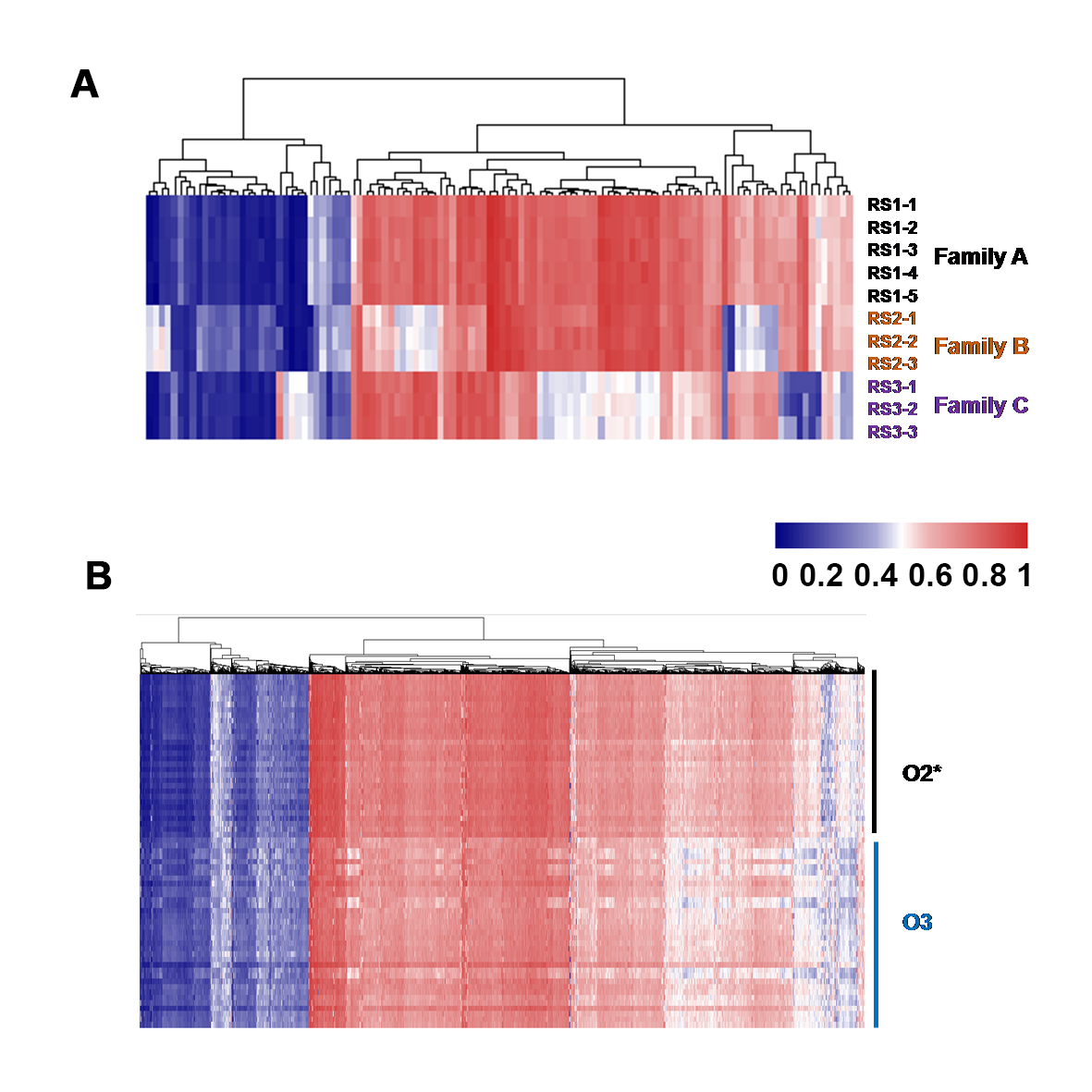

Supplement: S8 Fig — A) Heat map showing the family-specific DNA methylation sites on whole genome. B) Heat map showing the haplogroup O2* and haplogroup O3-specific DNA methylation sites on whole genome. Each vertical line represents a single site, with each row showing the β-value obtained in each individual tested. (TIFF) [file pone.0146402.s008.tiff]

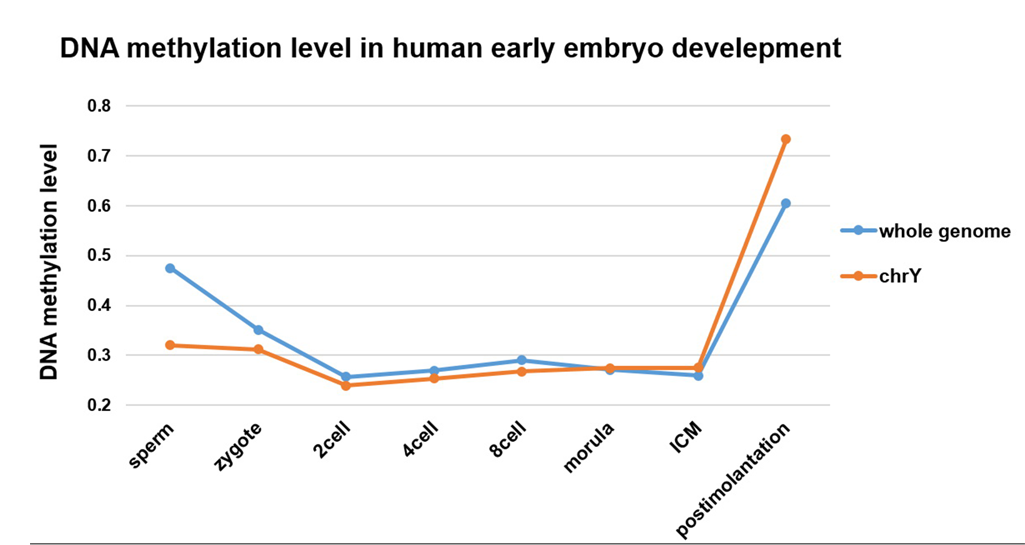

Supplement: S9 Fig — Published methylation data showing a de-methylation and then re-methylation process during early human embryonic development. Each data point represents the mean β-value of each stage. (TIFF) [file pone.0146402.s009.tiff]

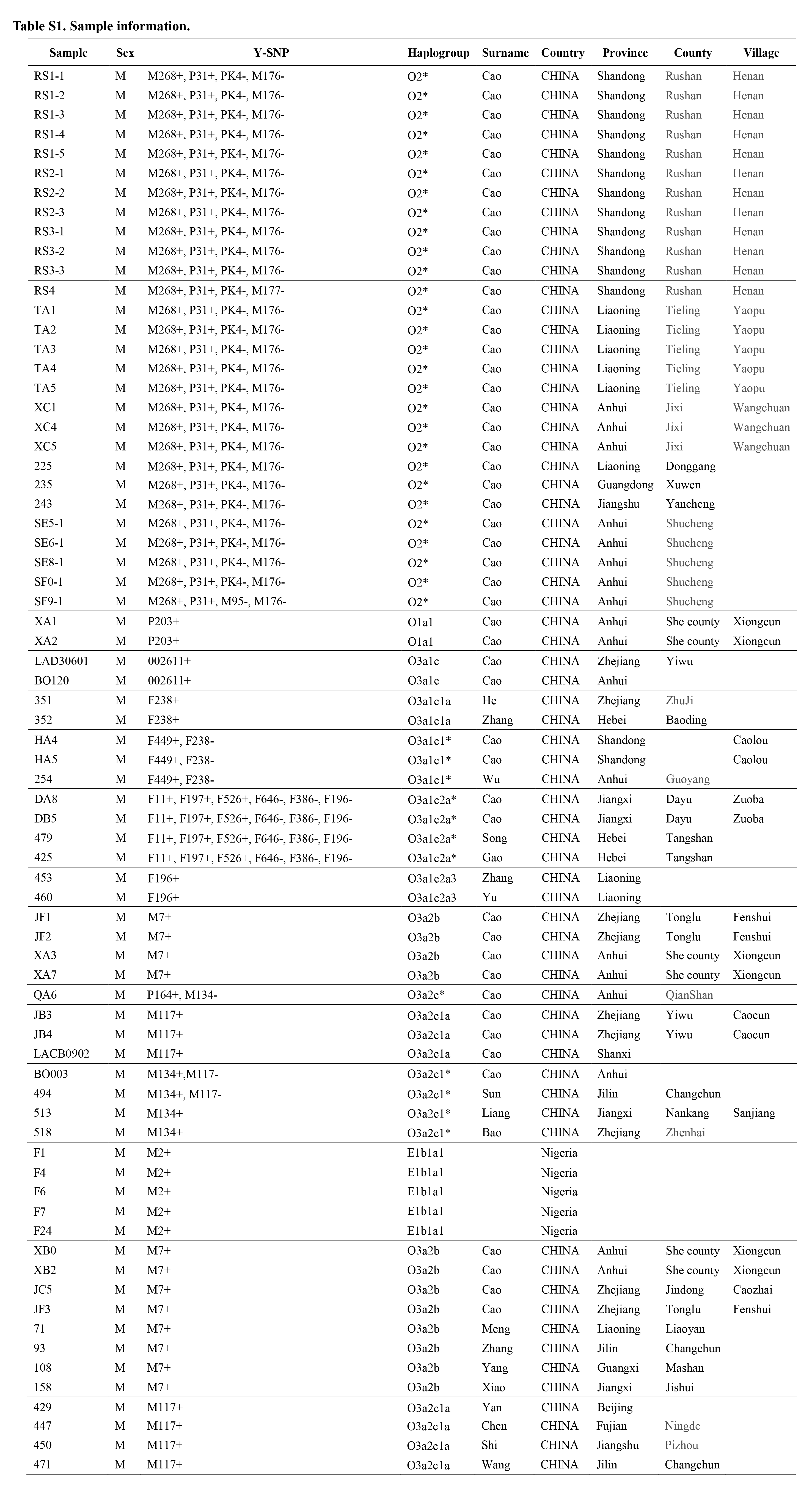

Supplement: S1 Table — (TIFF) [file pone.0146402.s010.tiff]

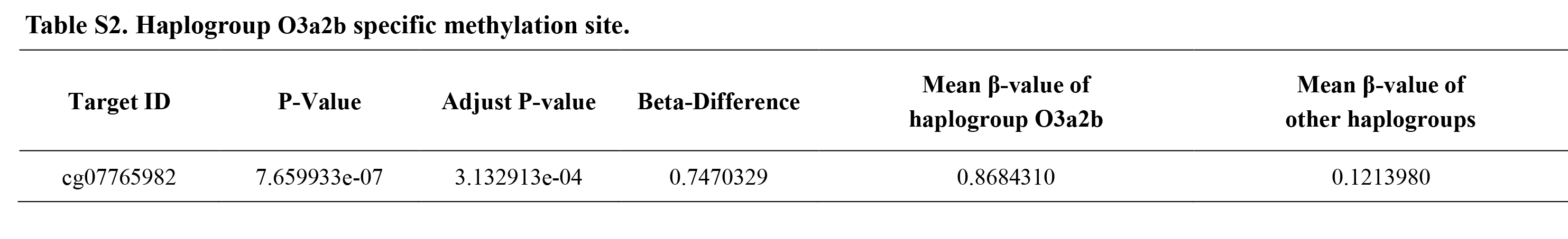

Supplement: S2 Table — (TIFF) [file pone.0146402.s011.tiff]

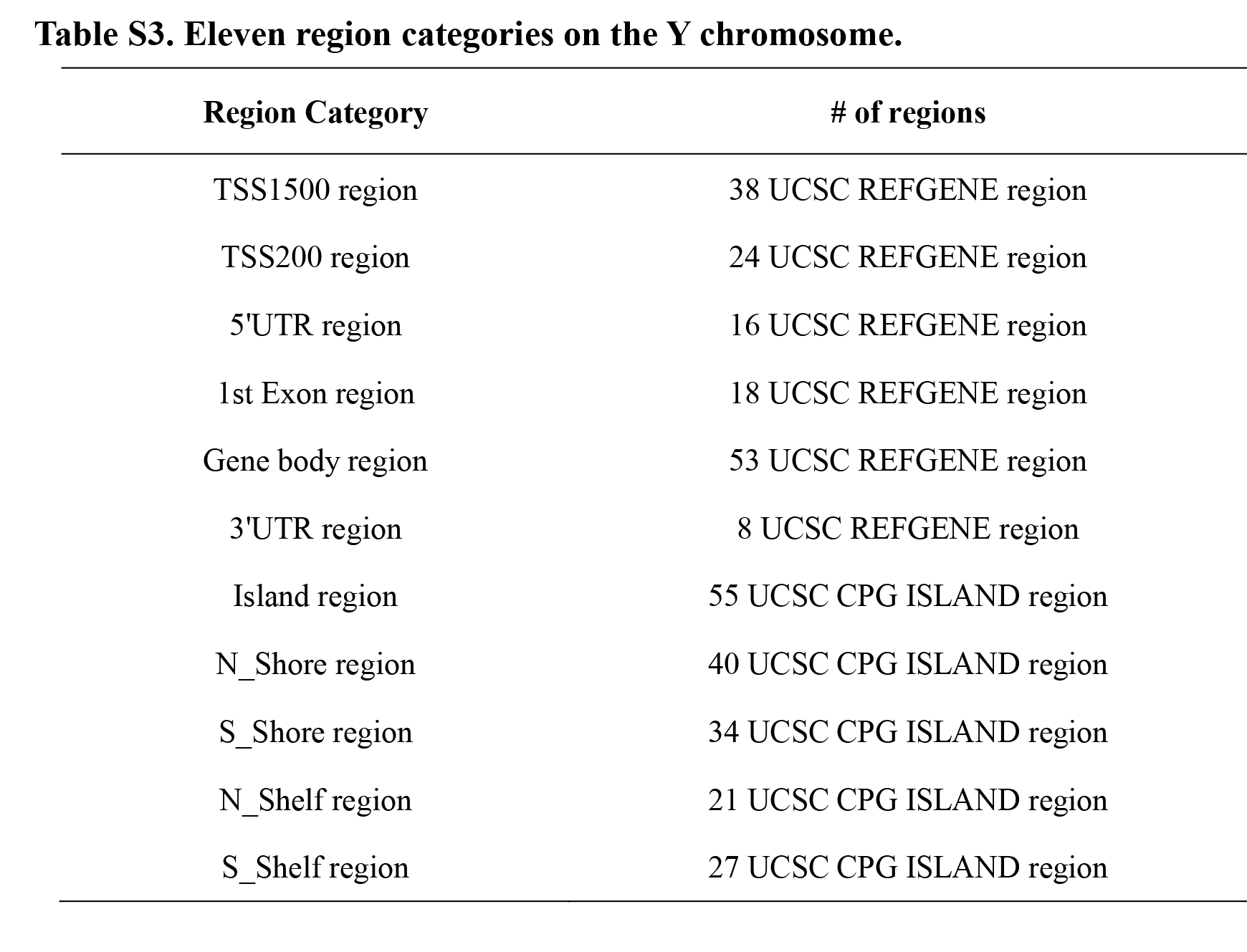

Supplement: S3 Table — (TIFF) [file pone.0146402.s012.tiff]

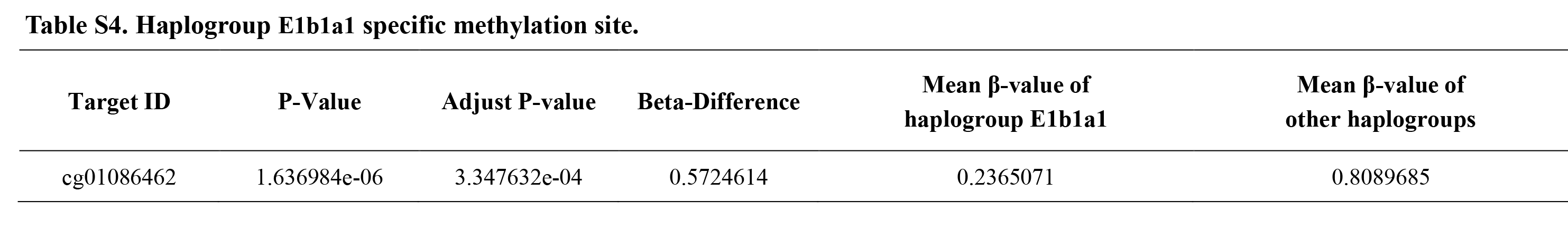

Supplement: S4 Table — (TIFF) [file pone.0146402.s013.tiff]
